# ELISA method to detect α-synuclein oligomers in cell and animal models

**DOI:** 10.1371/journal.pone.0196056

**Published:** 2018-04-26

**Authors:** Louise Berkhoudt Lassen, Emil Gregersen, Anne Kathrine Isager, Cristine Betzer, Rikke Hahn Kofoed, Poul Henning Jensen

**Affiliations:** University of Aarhus, DANDRITE – Danish Research Institute of Translational Neuroscience and Department of Biomedicine, Aarhus, Denmark; Louisiana State University Health Sciences Center, UNITED STATES

## Abstract

Soluble aggregates of α-synuclein, so-called oligomers, are hypothesized to act as neurotoxic species in Parkinson’s disease, Lewy body dementia and multiple systems atrophy, but specific tools to detect these aggregated species are only slowly appearing. We have developed an α-synuclein oligomer ELISA that allows us to detect and compare α-synuclein oligomer levels in different *in vivo* and *in vitro* experiments. The ELISA is based on commercially available antibodies and the epitope of the capture antibody MJF14-6-4-2 is folding- and aggregate-dependent and not present on monomers.

## Introduction

The aggregation prone protein α-synuclein (a-syn) is involved in Parkinson’s Disease (PD) as well as other α-synucleinopathies such as dementia with Lewy Body (DLB) and multiple system atrophy creating toxic aggregates within cells [1,2]. In healthy neurons, a-syn exists mainly as a monomeric protein in the neuronal presynapse, but within diseased cells, aggregation leads to the soluble oligomeric, and insoluble fibrillary forms of the protein. The finding of large aggregates of fibrillary a-syn in PD patients termed Lewy bodies [3] put the focus on the fibrillary state of a-syn for many years, but today oligomers are hypothesized to be the most cytotoxic form of a-syn [4–6].

What starts the oligomerization process and how the toxicity of the oligomers are handled in vivo remains to be elucidated. Furthermore, formation of oligomers in vitro reveals high heterogeneity with varying stability of the oligomers [7–10]. By the use of different methods using different additives, the comparison of recombinant oligomers between different research groups is not trivial. Oligomers might even exist as different species or strains that for example have different capability of uptake into cells, seeding, and hence toxicity as has been shown for certain types of fibrils [11]. In the pathogenic state, a-syn species (monomeric or oligomeric) accumulate in abnormal sites, which might lead to gain-of-function for other structures, new interaction partners, and/or potential loss of function of the normal monomeric-a-syn [12,13]. The unclear nature of oligomers makes it difficult to study their role in models and in disease. Aggregated forms of a-syn has been studied by denaturing methods like SDS-PAGE and immunoblotting that rely on the formation of SDS-resistant species. Non denaturing methods have been reported like dot blotting [14] and ELISA [15–19]. The development of aggregate specific ELISA methods that recognize neo-epitopes generated on a-syn aggregates are not trivial and only a few relying on mouse monoclonal antibodies have been reported [16–18], since our ELISA reported in 2009 was based on the polyclonal FILA antibody [15]. Brännström et al [18] screened for antibodies with a preference for aggregated a-syn but used monomer a-syn for immunization. Here we report on a novel ELISA which uses the novel monoclonal rabbit MJF14-6-4-2 antibody that *i*.) detects oligomeric a-syn, *ii*.) does not recognize monomeric a-syn, and *iii*.) can be used to detect a-syn oligomers in extracts from cells and tissues.

The ELISA detects oligomers in a wide range (0.25 ng/ml– 20.0 ng/ml) and allows easy detection of oligomers in cellular and transgenic animal models of α-synucleinopathies.

## Materials and methods

### Production of monomeric and oligomeric a-synuclein

Production of recombinant a-syn [20] and formation of a-syn monomers and oligomers was done according to previously described protocols [12] [21]. Oligomers was purified from monomers on a Superdex 200 10/30 column (GE healthcare), and protein concentration of the two fractions was measured by Bicinchoninic acid protein concentration assay (BCA). The size of eluted monomers and oligomers was calculated using standard protein weight markers (Sigma Aldrich, MWGF1000). Just after preparation, both oligomers and monomers aliquots were subjected to freezing in liquid nitrogen in normal non-coated Eppendorf tubes in high concentration (150 μg/ml) and low volume (20 μl). For each ELISA, one aliquot was thawed and then discarded afterwards.

### Antibodies

The MJF-14-6-4-2 antibody was generated in a collaboration between our laboratory, Epitomics, and the Michael J. Fox Foundation, and marketed by Abcam.

Polyclonal rabbit anti-a-syn, ASY-1 [22] and the a-syn aggregate specific FILA-1 antibodies [23], also produced in house, are previously described. Mouse monoclonal anti-a-syn (BD 610787) was purchased from BD Transduction Laboratories.

### Enzyme linked immunosorbent assay (ELISA)

#### Oligomer ELISA

96-well Maxisorp plates were incubated with 100 μl 62.5 ng/ml MJF-14-6-4-2 in ELISA coating buffer (0.1 M NaHCO_3_ in phosphate buffered saline PBS (2.8 mM NaH_2_PO_4_, 7.2 mM Na_2_HPO_4_, 123 mM NaCl), pH = 9.5) overnight. The plate was washed three times with TBS-tween (1.5M NaCl, 200 mM Tris, 0.05% Tween-20, pH 7.4) and ELISA blocking buffer (PBS + 10% fetal calf serum (FCS)) added for 2 hours at room temperature (RT). After washing, samples were diluted in PBS in a total volume of 100 μl (for tissue samples, 2 μg of total protein—for cell line samples 4 μg of total protein was added). The following day, wells were washed, and 100 μl 0.5 μg/ml mouse monoclonal anti-a-syn (BD Transduction Laboratories, 610787) was added for 2 hours. After washing, anti-mouse-HRP antibody was added for 1.5 hours followed by wash and addition of 100 μl 3,3’,5,5’-Tetramethylbenzidine (TMB) to each well. Color detection is followed every 5 minutes by measuring the absorbance at 650 nm, and at the desired amount of color detection for the samples in question, the enzymatic reaction is stopped by the addition of 100 μl 1M phosphoric acid. The absorbance is measured at 450 nm and compared to signals for oligomer standards. Samples were always added in duplicates or triplicates. Coefficient of variance (CV) was calculated by dividing the standard deviation by the duplicate mean.

#### A-syn total ELISA

Follows the same protocol as the oligomer ELISA except that the ASY-1 antibody [22] was used as the capture antibody in the concentration 99.6 ng/ml, and monomeric a-syn was used as standard.

#### Direct ELISA

96-well Maxisorp plates were incubated with 100 μl 20 ng/mL freshly prepared oligomer in PBS overnight at RT. The plates were blocked in ELISA blocking buffer for 2 hours at RT and washed three times in TBS-tween. Next, 100 μl 15.6 ng/mL MJF-14-6-4-2 pre-incubated overnight at RT with alpha-synuclein monomer or oligomer in TBS-tween were added to the wells and incubated for 2 hours at RT. After washing and incubation with anti-rabbit-HRP antibody for 30 min at RT, detection by TMB follows the same steps as the oligomer ELISA above.

### Synaptosomal lysates from ASO mouse brain

Brains were collected from ASO male mice (age 7–8 months) or control wild type mice (12 months) and immediately homogenized in 5 x volume of ice cold homogenization buffer (320 mM sucrose, 4 mM HEPES-NaOH, 2 mM EDTA, complete protease inhibitor mix (Roche), phosphatase inhibitors (25 mM β-glycerolphosphate, 5 mM NaF, 1 mM Na_3_VO_4_) pH 7,4) by 20 strokes in a loose-fitting glass-homogenizer. The resulting homogenate was centrifuged at 1,000 g, 10 min, 4° C, and the pellet discarded. The supernatant was centrifuged at 12,000 g, 15 min, 4° C, and the pellet resuspended in 1 ml of homogenization buffer and exposed to another centrifugation of 12,000 g, 15 min, 4° C. The resulting pellet enriched for synaptosomes [24] (herein referred to as synaptosomes) was extracted with RIPA buffer (50 mM Tris (pH 7,4), 159 mM NaCl, 1% Triton X-100, 2 mM EDTA, 0.5% sodium deoxycholate, 0.1% SDS) for 1 hour, where after samples were spun 25,000 g, 20 min, 4° C. Protein concentration was measured using the bicinchoninic acid assay and 2–4 μg of total protein was added to each well in both the oligomer ELISA and the total a-syn ELISA.

Mice were hold under temperature constant conditions with a 12-hour light/dark cycle with constant water and food available. All mice experiments were performed according to the Danish Animal Welfare Agency (approval number 2017-15-0201-01203) and according to national and international guidelines for the care and use of animals. Mice were sacrificed via sedation followed by perfusion to get rid of blood from tissues.

### Dot blot

Dot blot was used for validation of the binding of the conformational specific antibody to oligomers, and for formic acid denaturation of ASO mouse brain synaptosomal extracts. Samples were spotted directly onto a nitrocellulose membrane using vacuum. The membrane was incubated with primary (anti-a-syn (BD), ASY-1, or FILA-1) and secondary horseradish peroxidase (HRP) conjugated antibodies. Proteins were visualized using ECL in a Fuji LAS-300 Intelligent Dark Box (Fujifilm, Japan).

### Cell culture

Cells were maintained at 37° C and 5% CO_2_. SH-SY5Y cells stably expressing tet-Off inducible a-syn were kindly provided by Leonidas Stefani and Kostas Vekrellis [25]. Before differentiation, cells were grown in RPMI medium supplemented with 15% fetal calf serum (FCS) and 50 μg/ml penicillin/streptomycin. Doxycycline (1 μg/ml) was added to suppress expression of a-syn. Cells were differentiated by 20 μM retinoic acid. Doxycycline treatment was stopped two days after onset of differentiation. Experiment was stopped after 5 or 10 days of expression by extracting with RIPA buffer on ice for 30 minutes followed by centrifugation at 25,000 g, 20 min, 4°C. Protein concentration was measured in the supernatant and 4 μg was added to each well in the oligomer ELISA or the total a-syn ELISA. All cells were tested for mycoplasma.

### Statistics

Results were compared using the Student’s unpaired t-test when comparing two groups and one-way ANOVA followed by Turkey’s *post hoc* test for multiple comparison. Data reaching a p-value lower than 0.05 are considered statistically significant. Significance is marked with an asterisk: *p<0.05; **p<0.005.

## Results

We have developed a sandwich-type α-synuclein (a-syn) oligomer ELISA with the use of the conformational and aggregate specific rabbit monoclonal antibody MJF14-6-4-2 (Abcam) as capture antibody. The mouse monoclonal anti-a-syn antibody (BD 610787) is used as the detection antibody followed by amplification with anti-mouse horseradish peroxidase and detection with 3,3’,5,5’-Tetramethylbenzidine (TMB).

**Fig 1:**
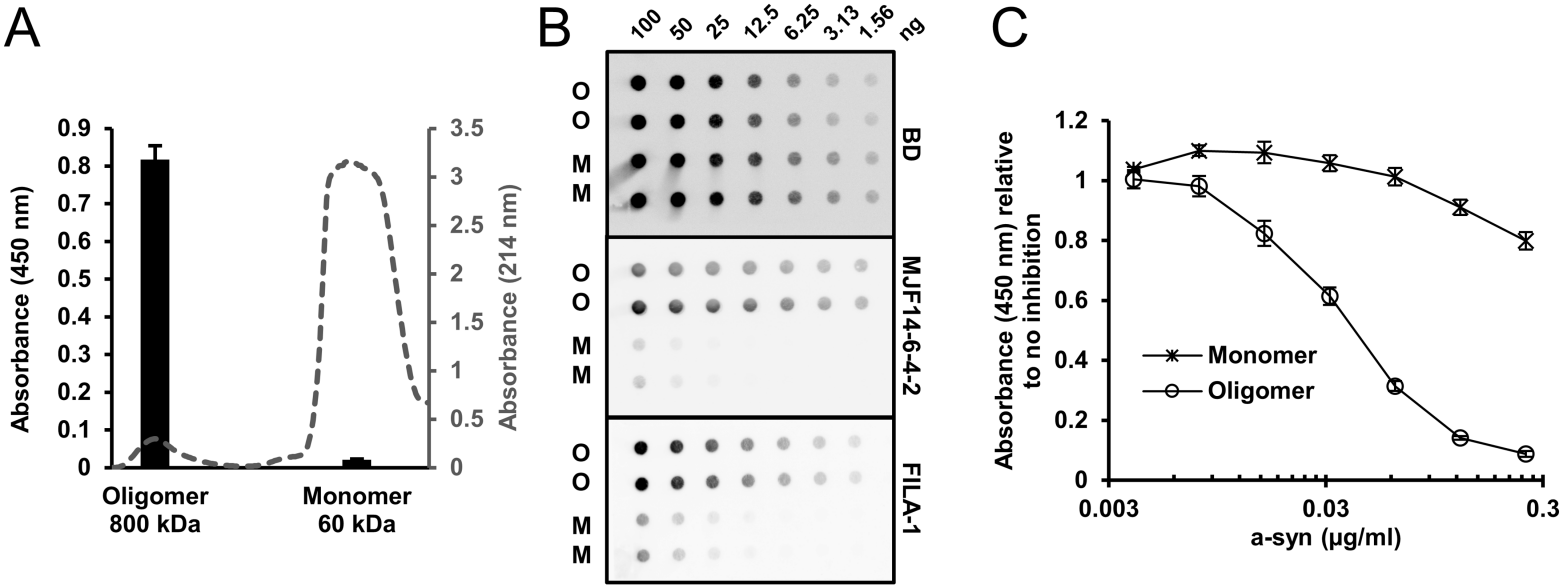
Specificity and affinity of the MJF14-6-4-2 antibody. **A.** Monomers and oligomers were isolated by gel filtration and eluted in two distinct fractions with peak values corresponding to 60 kDa and 800 kDa, respectively (grey broken line). The absorbance profile at 214 nm is indicated at the right ordinate axis. To validate the specificity of the oligomer ELISA we incubated antibody coated wells with the same concentration of monomer and oligomer (9 ng/mL) and used a monoclonal anti-a-syn antibody as second antibody. Bound antibody was detected by anti-mouse HRP and the TMB color change was measured as absorbance at 450 nm. The ELISA gives a strong signal for oligomers whereas there is only a negligible signal from the monomer sample (black bars). Background absorbance was subtracted. The ELISA signal (absorbance at 450 nm) is presented at left ordinate axis. **B.** We compared the aggregate specificity of the MJF14-6-4-2 antibody with the well characterized polyclonal antibody FILA-1 using dot blotting analysis. Dilutions of monomers (M) and oligomers (O) were immobilized and tested for their antibody binding. The non-conformation dependent monoclonal a-syn antibody (BD) (0.5 μg/ml) demonstrated equal loading of monomer and oligomer. MJF14-6-4-2 (Abcam) (2.2 ng/ml) and FILA-1 (3.5 μg/ml) antibodies display the same extend of selectivity for the aggregated a-syn. **C.** The affinity of MJF14-6-4-2 antibody was assessed by a direct ELISA on oligomer coated wells. The MJF14-6-4-2 (15.6 ng/ml) antibody was pre-incubated overnight with increasing concentrations of monomer or oligomer before being applied to the oligomer containing wells. The concentration of oligomer that gave 50% inhibition was measured to be 42 ng/ml. The K_D_-value of MJF14-6-4-2 was calculated to be 2.9 · 10^−10^ M based on the assumption that 10 a-syn monomers are required to generate a folding specific MJF14-6-4-2 epitope. Both A and C show one representative experiment of 3. Standard deviations represent three technical replicates.

The a-syn monomers and oligomers used for the set-up of the ELISA were prepared and isolated by gel filtration according to previously published protocols and could be completely depolymerized into monomers by denaturants [9,21]. Fig 1A displays the gel filtration profile when separating monomer (60 kDa) from oligomers (800 kDa). When analyzing these two fractions, the oligomer yields a clear signal on the oligomer ELISA, while only a negligible signal is detected in the monomer fraction (Fig 1A). The aggregate specific binding of MJF14-6-4-2 was validated using dot blotting that represents a non-denaturing method for studying protein epitopes. The loading of equal amounts of monomer and oligomer a-syn was validated using the a-syn antibody (BD) that we also use as detection antibody in the ELISA. Fig 1B demonstrates a similar dilution of monomer and oligomer α-synuclein on the membrane by binding of BD antibody. By contrast MJF14-6-4-2 at a concentration of 2.2 ng/ml detected the oligomer efficiently with only a weak binding to the highest concentrated monomer dot with 100 ng immobilized. To validate the aggregate conformation-specific nature of the oligomers we also tested the membranes with the established FILA-1 antibody using a concentration of 3.5 μg/ml, and this demonstrated a similar binding as MJF14-6-4-2 [26]. Hence the MJF14-6-4-2 antibody is aggregate specific as FILA-1 but exhibits a much higher affinity as demonstrated by the more than 1000-fold lower concentration used to generate the signals on the dot blot. To assess the affinity of the MJF14-6-4-2 antibody towards a-syn oligomers, we established a direct ELISA with a-syn oligomer coated wells (Fig 1C). Increasing concentrations of oligomers pre-incubated with MJF14-6-4-2 inhibited the detection of coated oligomer, whereas a-syn monomer only had minimal effect in the applied concentrations. K_D_ was determined as described by Neri et al. [27] to 2.9*10^−10^ M based on the concentration of oligomer at 50% inhibition, and the assumption that 10 monomers are required to generate a folding specific MJF14-6-4-2 epitope.

**Fig 2:**
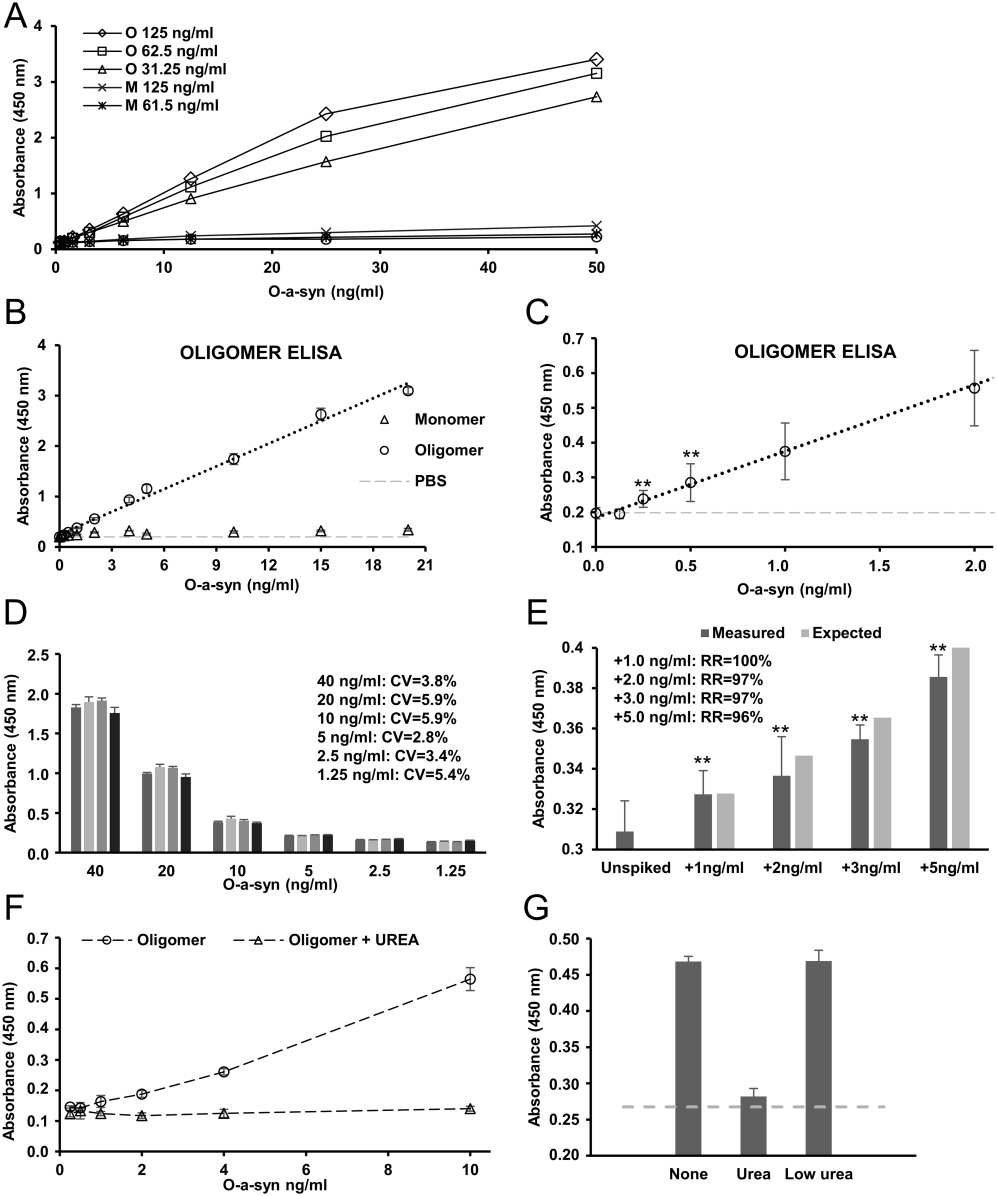
A-syn oligomer ELISA sensitivity and specificity. **A.** Test of three different concentrations (0.125, 0.0625, and 0.03125 μg/ml) of capture antibody MJF14-6-4-2 (MJF) for monomeric and oligomeric a-syn for the oligomer ELISA. 0.0625 μg/ml is chosen as the optimal concentration for MJF14-6-4-2 based on sensitivity and specificity from monomeric a-syn. **B.** The oligomer ELISA detects oligomeric a-syn in a linear range from 0.25–20 ng/ml oligomer a-syn with the use of MJF14-6-4-2 as capture antibody and the anti-a-syn (BD) as detection antibody (R^2^ = 0.99). Monomeric a-syn is not detected by the oligomer ELISA in this range. Negative control (PBS) shown in light grey. **C.** The graph from C. zoomed in on the lower part of the X-axis. The oligomer ELISA detects down to 0.25 ng/ml oligomeric a-syn (P = 0.001). **D.** Determination of inter-assay coefficient of variation (CV). Comparison of results from four different runs is used to determine CV for the oligomer ELISA. CV is calculated for 6 different concentrations of oligomers (1.25 ng/ml– 40 ng/ml) resulting in 6 different CVs values all below 6%—in average 4,5%. **E.** Recovery by recombinant a-syn oligomers spiked into WT mouse brain synaptosomes. Mouse brain synaptosomes are spiked with different amounts of oligomers (1, 2, 3, and 5 ng/ml). Expected increase in absorbance is calculated from an internal series of controls, and the recovery rate is calculated for each concentration of spiked amount of oligomer. All recovery rates are above 95%—in average 98%. Data are an average of 4 different experiments run in triplicates. Data are compared to unspiked for significance. **F.** Validation that a-syn oligomer signal is caused by conformational epitopes. Treatment with 8M urea inhibits the a-syn oligomer signal on the oligomer ELISA. Higher concentrated oligomers are treated for 3 hours with 8M urea, and then diluted to 3mM urea before addition to the ELISA. **G.** To validate that the urea treatment does not affect the binding capability of the ELISA or/and the antibodies used, the experiment was run with longer incubation time, an oligomer concentration of 2.5 ng/ml, and low amounts of urea equivalent to the amount of UREA present in the urea treated oligomers after dilution (urea concentration during running conditions = 3 mM). 8M urea treatment reduces the oligomer signal, whereas 3mM urea treatment does not remove the oligomer signal. “None” represents non-treated a-syn oligomers, whereas background signal from PBS without a-syn is shown by the dotted grey line. Both G. and H. show one representative experiment of 3. Standard deviations represent three technical replicates. Significance for all parts of figure is marked with an asterisk: *p<0.05; **p<0.005. Results were compared using one-way ANOVA followed by Turkey’s *post hoc* test for multiple comparison.

A concentration of 62.5 ng/ml capture antibody MJF14-6-4-2 was identified as the optimal for coating the wells based on sensitivity and background signal from a-syn monomers (Fig 2A). Based on this coating concentration, the oligomer ELISA detected oligomeric a-syn in a linear range between 20 ng/ml and 0.25 ng/ml. The signal from monomeric a-syn was negligible in this range (Fig 2B and 2C).

To determine the inter-assay coefficient of variance (CV), we compared the measurement of a serial dilution of oligomeric a-syn between four different measurements. Careful handling of oligomers is important for the assay as for example multiple freezing and thawing cycles will remove the MJF14-6-4-2 epitope on the oligomers in agreement with the reversible nature [21]. The temperature and detection time can also cause variable results. With the pernickety handling of oligomers and a development time of 30 minutes, an inter-assay CV of 4.5% (average) was obtained (Fig 2D). To calculate the intra-assay CV, we compared triplicates from six different concentrations ranging from 1.25 ng/ml to 40 ng/ml of oligomeric a-syn in four different plates. Of the 72 measurements, we found an average intra-assay CV of 4.5% with the maximal CV being 5.9%. However, the inclusion of a serial dilution of standard samples in each run is highly recommended, as it also opens the possibility for the researcher to choose the best or multiple detection times for the samples in question.

To demonstrate the ELISA could recover in vitro generated oligomeric a-syn we spiked increasing amounts into detergent solubilized brain synaptosomes from wild type mouse. Fig 2E demonstrates we were able to recover >95% of the added a-syn oligomers, when added in concentrations of 1–5 ng/ml.

Finally, we wanted to demonstrate that the ELISA signal is indeed aggregation and conformational specific. To denature the oligomers, we treated the oligomeric a-syn stock solution (150 μg/ml) with 8M urea for 3 hours at room temperature before diluting it more than 1000-fold prior to testing on the oligomer ELISA. This treatment completely inhibited the oligomer specific signal (Fig 2F). To ensure the effect was not caused by the trace amounts of urea in the diluted sample we compared the oligomer signal in the ELISA by oligomers incubated with 8 M urea before being diluted to 3 mM urea with oligomers directly being diluted in 3 mM urea. Fig 2G demonstrates that the presence of 3 mM urea did not affect the oligomer signal compared to the complete removal by 8 M urea. Hence, our oligomer signal is dependent on a folding specific epitope sensitive to denaturing agents.

**Fig 3:**
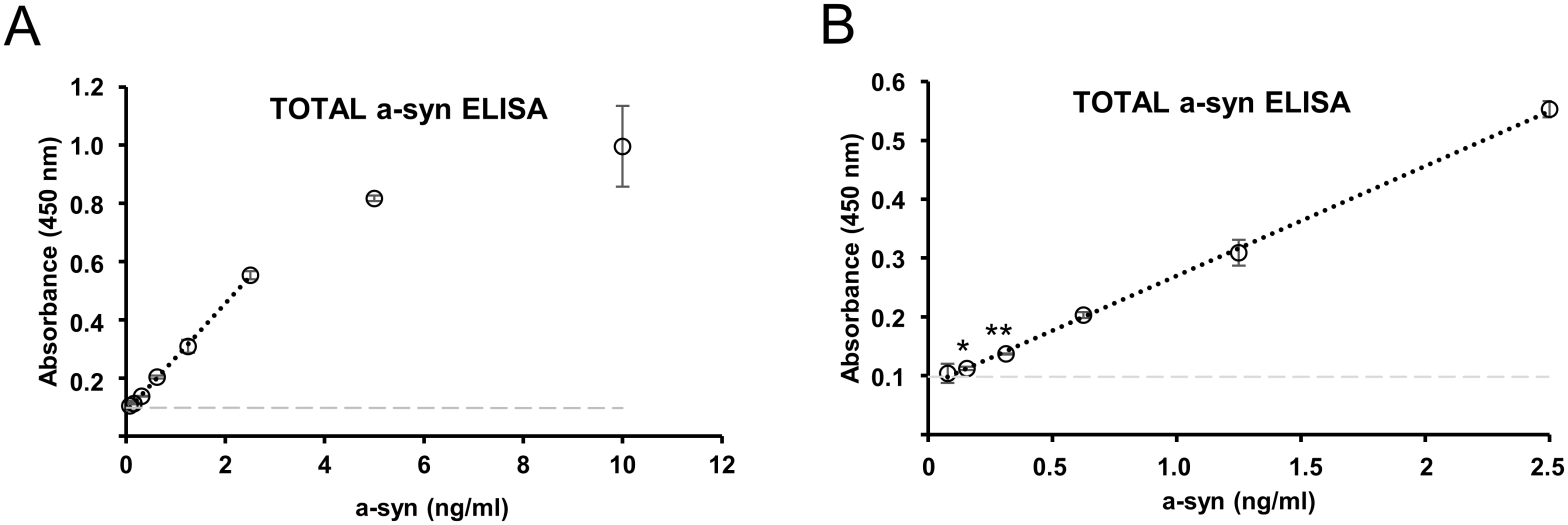
Validation of total a-syn level by a total a-syn ELISA. **A.** Standard a-syn ELISA is used as control with our in-house ASY-1 antibody as capture antibody. The ASY-1 antibody detects monomeric a-syn in linear range up to 2.5 ng/ml (R^2^ = 0.99). The detection is saturated at 10 ng/ml. **B.** The graph from E. zoomed in on the lower part of the X-axis. The ASY-1 ELISA detects down to 0.16 ng/ml of monomeric a-syn (P = 0.02). Negative control (PBS) shown in light grey.

In order to relate the levels of oligomeric a-syn to total a-syn, we also developed a total a-syn ELISA using ASY-1 [22] that detects total a-syn from 0.16 ng/ml (Fig 3A). This total a-syn ELISA detects in a linear range up to 2.5 ng/ml (R^2^ = 0.99) (Fig 3B). Recombinant monomeric a-syn is used for the serial standard curve shown in Fig 3.

**Fig 4:**
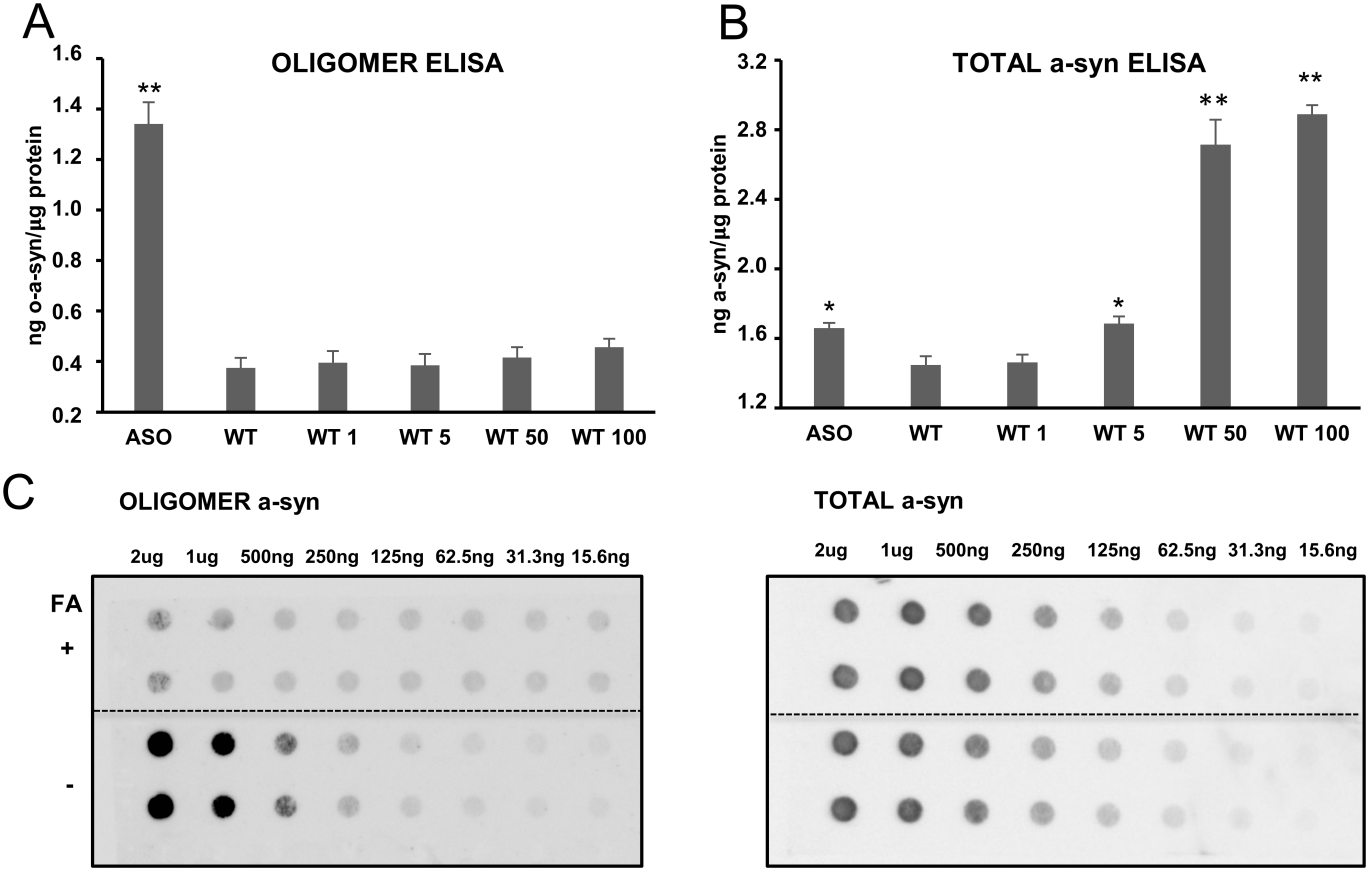
Detection of oligomeric a-syn in synaptosomes isolated from whole brains of mThy-h-a-syn transgenic mice (ASO) and verification of folding dependent epitopes. Oligomer a-syn (A.) and total a-syn (B.) levels are measured in whole brain synaptosomes from adult ASO (n = 3) and WT (n = 3) mice that were extracted in detergent containing RIPA buffer and analyzed by ELISA for their a-syn content. **A**. The oligomer a-syn ELISA reveals increased oligomer levels in the ASO mice compared to the WT mice. The oligomer signal is aggregate specific because no increase is obtained by spiking the sample with increasing amounts of recombinant monomer standards **B**. The total level of a-syn is increased in ASO mice compared to WT and spiking the sample with the same recombinant monomeric a-syn standard as used in panel A increases the level of total a-syn when more than 5 ng/ml monomer is added. **B**. Bars in A and B represents standard deviation of three biological replicates (ASO and WT). Significance is marked with an asterisk: *p<0.05; **p<0.005 and indicate in (A.) and (B.) the given sample related to wt. Results were compared using one-way ANOVA followed by Turkey’s *post hoc* test for multiple comparison. **C.** Folding specificity of the MJF14 binding to a-syn species in synaptosomes was analyzed by dot blotting in the absence and presence of denaturing 50% formic acid (FA). Dilution of RIPA extracts of ASO mouse brain synaptosomes was applied to the dot blots in the absence and presence of treatment with denaturing 50% formic acid (FA). The filters were tested for binding of MJF14-6-4-2 to detect the aggregate dependent epitope (left panel-oligomer) and ASY-1 to detect total a-syn (right panel-total a-syn). Right dot blot shows the total level of a-syn, which is equal in the two samples treated or untreated with FA. FA treatment removes the epitope for the MJF14-6-4-2 antibody but not the ASY-1 epitopes confirming the folding specificity of the MJF14-6-4-2 epitope.

To test the oligomer ELISA in a complex sample, we determined the amounts of a-syn oligomers present in detergent extracts of whole brain synaptosomes of adult ASO mice overexpressing a-syn under the Thy1 promoter, which display an early motoric phenotype [28,29]. The total amount of a-syn was increased in the ASO mouse (n = 3) compared to wild type littermates as reported [28] (Fig 4B), but also the amounts of oligomers were increased (Fig 4A). To validate that the oligomer signal in the ELISA was not a false positive signal caused by the increased level of total a-syn, we spiked increasing amounts of monomer a-syn in samples from WT mice (n = 3). The total a-syn ELISA readily detected the addition of more than 5 ng/ml monomer a-syn, whereas the signal in the oligomer ELISA was not increased by adding up to 100 ng/ml demonstrating the signal in the oligomer ELISA is not caused by increased total a-syn levels (Fig 4A and 4B). To validate that the MJF14-6-4-2 signal in the detergent extracts of ASO brain synaptosomes were represented by a folding specific epitope, we analyzed the effect of denaturing the sample with 50% formic acid for 30 min before applying the sample onto the filter prior to dot blotting. Fig 4C demonstrates that formic acid treatment reduces the binding to background levels compared to the strong signal in the untreated sample that allows detection in as low as 250 ng total synaptosomal protein. To control that the formic acid treatment did not inhibit the binding of a-syn to the membrane, we demonstrate that the ASY-1 binding to the synaptosomal a-syn was unaffected by the formic acid treatment and can detect total a-syn in samples containing more than 125 ng synaptosomal protein.

**Fig 5:**
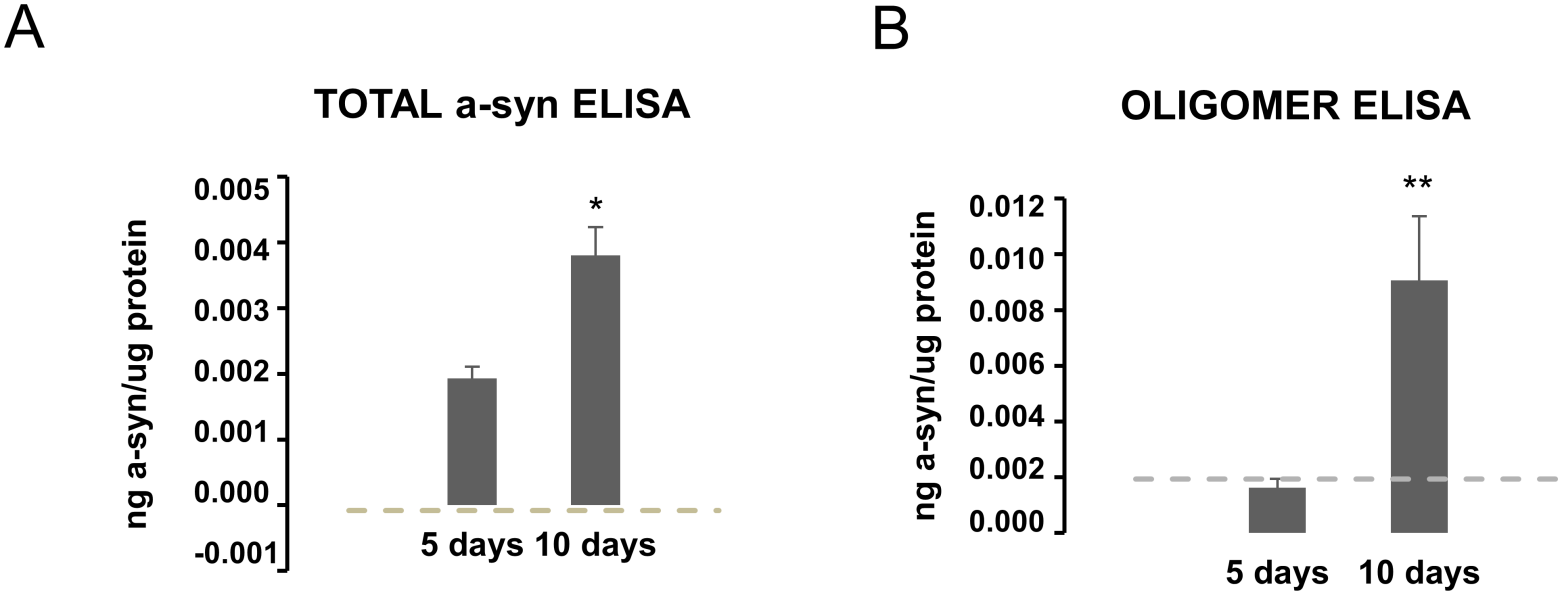
Time dependent development of a-syn oligomers in a non-mitotic a-syn transgenic cell model. A-syn SH-SY5Y Tet-off cells were differentiated with retinoic acid to induce a non-mitotic state where after a-syn expression was induced by removal of doxycycline. PBS background is shown by the dotted grey line. **A.** Measurement of a-syn by the total a-syn ELISA in RIPA extracts demonstrated a linear increase in cellular a-syn that demonstrates approximately a doubling of a-syn from day 5 to day 10. **B.** Analysis of the RIPA extracts by the oligomer ELISA demonstrates no detectable oligomers after 5 days but a significant increase after 10 days. Bars represents standard deviation of 3 biological experiments (all run in three technical replicates). The Student’s unpaired t-test was used to compare 5 and 10 days. Significance is marked with an asterisk: *p<0.05; **p<0.005. For A., 5 days is also significantly different from PBS background, whereas 5 days in B. cannot be distinguished by PBS.

The retinoic acid differentiated WT a-syn SH-SY5Y cell model with Tet-off inducible expression displays degenerative changes after 6 days of a-syn expression when compared to cells without a-syn expression and the degeneration can be attenuated by the aggregation inhibitor Scyllo-inositol [25]. Using the same model, we confirmed the progressive increase in cellular a-syn from 5 to 10 days of a-syn expression by the total a-syn ELISA (Fig 5A) and demonstrated by the oligomer ELISA that the oligomer signal develops later than day 5 with a strong signal after 10 days (Fig 5B). This development of oligomer signal after day 5 corresponds to the period wherein the models start to develop enhanced cell death [25].

## Discussion

It is evident that α-synuclein (a-syn) is closely linked to PD and LBD but it is still unclear how a-syn aggregates are mechanistically linked to neuronal degeneration and what the nature of the toxic species are. A commonly accepted hypothesis is that these species are soluble and thus able to get access to various parts of neurons more easily than fibrillar amyloid-type aggregates that are deposited in intracellular inclusions like Lewy bodies and Lewy neurites [30].

The ability to measure soluble oligomers has been impeded by the lack of tools that can discriminate between native and aggregated species. The first generation of ELISA-assays were built on the principle of using two identical monoclonal a-syn antibodies that were unable to bind monomers [19]–this assay was used in cerebrospinal biofluids. The next generation was based on the use of conformational specific antibodies recognizing epitopes developed on aggregates that are missing on native putatively unfolded a-syn. A polyclonal rabbit antibody FILA-1 was used and detected elevated levels in brain extracts of DLB brain compared to controls [15]. This strategy has been optimized by the use of monoclonal antibodies like our rabbit monoclonal MJF14-6-4-2 and the mouse monoclonal Syn-O2, which displays preferential binding to oligomeric α-synuclein aggregates and holds promise in CSF biomarker studies [16]. Also, a mouse monoclonal antibody developed against a-syn oligomers was used by Fagerqvist et al to show increased levels in a-syn A30P transgenic mice compared to wt using a homopaired ELISA [17]. We now report on the recently developed high affinity rabbit monoclonal antibody MJF-14-6-4-2 that binds to an epitope on both filamentous and oligomeric a-syn. This MJF14-6-4-2 epitope is present in human brain tissue [31] and in symptomatic a-syn transgenic mice [29]. We demonstrate that the epitope is lost when in vitro-formed oligomers and oligomers present in synaptosome extracts of ASO transgenic mice are denatured prior to analysis. Such a denaturation-sensitive aggregate-specific epitope is likely not present on putative physiological a-syn polymers as those proposed by the Selkoe laboratory in erythrocytes [32] and by Burré on presynaptic membranes [33]. Rather, it arises from specific structural conformations that is only present on disease-associated aggregates as corroborated by deuterium exchange mass spectrometry [21].

The sensitivity of the oligomer ELISA towards in vitro-formed oligomers was 0.25 ng/ml a-syn, and the signal was completely blocked if the oligomers were denatured by 8 M urea prior to being diluted into the assay buffer. Our data demonstrates that caution has to be exerted when attempting to quantify oligomers in biological extracts by the use of in vitro formed oligomer standards. Fig 3A and 3B demonstrate that the level of oligomers is within the same order of magnitude as the total a-syn measured by our total ELISA. Quantitative analyses of the fraction of total a-syn that is oligomerized is not well studied but oligomeric a-syn has in CSF been reported to account for only approximately 5–10% of total a-syn [16]. The potential overestimation of biological oligomers may be due to a better presentation of the aggregate specific epitopes on cell-derived oligomers, e.g. due to binding of metabolites or proteins [12], compared to in vitro formed oligomers but can also be due to the labile nature of our non-stabilized oligomer standard [21].

We were able to detect a significant signal in 2 μg of total protein from detergent extracts of brain membrane extracts from ASO transgenic mice enriched for synaptosomes. The signal was simply not due to the presence of elevated levels of transgenic a-syn because spiking of up to 100 ng/ml of monomeric a-syn into the extract had no effect on the signal. The oligomer ELISA thus contributes a qualitative structural characteristic to the detected a-syn, which contrast the ELISA used to detect spreading of a-syn pathology in the prion-like M83 mice model [34]. The MJF14 ELISA is, to the best of our knowledge, the first reported to demonstrate development of a-syn aggregates in a cell model. Here the conformational specific MJF14-6-4-2 antibody clearly demonstrates that its epitope first develops in the cell after 10 days, whereas there is already significant a-syn expression after 5 days. The development of MJF14-6-4-2 positive oligomers correlates with the cell death in this SH-SY5Y model that first demonstrates toxicity after 6 days, where after it progresses over time [25].
